# Statistical Quantification of Methylation Levels by Next-Generation Sequencing

**DOI:** 10.1371/journal.pone.0021034

**Published:** 2011-06-15

**Authors:** Guodong Wu, Nengjun Yi, Devin Absher, Degui Zhi

**Affiliations:** 1 Department of Biostatistics, University of Alabama at Birmingham, Birmingham, Alabama, United States of America; 2 HudsonAlpha Institute for Biotechnology, Huntsville, Alabama, United States of America; Max Planck Institute for Evolutionary Anthropology, Germany

## Abstract

**Background/Aims:**

Recently, next-generation sequencing-based technologies have enabled DNA methylation profiling at high resolution and low cost. Methyl-Seq and Reduced Representation Bisulfite Sequencing (RRBS) are two such technologies that interrogate methylation levels at CpG sites throughout the entire human genome. With rapid reduction of sequencing costs, these technologies will enable epigenotyping of large cohorts for phenotypic association studies. Existing quantification methods for sequencing-based methylation profiling are simplistic and do not deal with the noise due to the random sampling nature of sequencing and various experimental artifacts. Therefore, there is a need to investigate the statistical issues related to the quantification of methylation levels for these emerging technologies, with the goal of developing an accurate quantification method.

**Methods:**

In this paper, we propose two methods for Methyl-Seq quantification. The first method, the Maximum Likelihood estimate, is both conceptually intuitive and computationally simple. However, this estimate is biased at extreme methylation levels and does not provide variance estimation. The second method, based on Bayesian hierarchical model, allows variance estimation of methylation levels, and provides a flexible framework to adjust technical bias in the sequencing process.

**Results:**

We compare the previously proposed binary method, the Maximum Likelihood (ML) method, and the Bayesian method. In both simulation and real data analysis of Methyl-Seq data, the Bayesian method offers the most accurate quantification. The ML method is slightly less accurate than the Bayesian method. But both our proposed methods outperform the original binary method in Methyl-Seq. In addition, we applied these quantification methods to simulation data and show that, with sequencing depth above 40–300 (which varies with different tissue samples) per cleavage site, Methyl-Seq offers a comparable quantification consistency as microarrays.

## Introduction

DNA methylation is an epigenetic regulatory mechanism implicated with various human diseases [1], [2]. cytosine nucleotides in DNA molecules, primarily in the CpG context, may be methylated, and the changes in DNA methylation status can modulate expression levels of genes [3], [4], [5], [6], [7] and therefore phenotype [8], [9], [10], [11].

In the past, measurement of DNA methylation was only feasible and affordable for a small number of individuals at a limited number of sites. Recently, genome-scale technologies have been developed for profiling DNA methylation status of individuals, including sequencing-based technologies that can survey DNA methylation levels genome-wide with base-pair resolution [12], [13], [14].

With the advancement of sequencing technology, the cost of large-scale sequencing has dropped considerably. Therefore, genome-wide epigenetic association studies may soon become feasible in large cohorts. At present, however, genome-wide sequencing of methylation is most economical when the DNA samples are first enriched with target regions by genome partition techniques. There are a number of such technologies available to investigators. See recent reviews [12], [13] for the experimental aspects of these technologies. In this work, we focus on Methyl-Seq [15] and RRBS [16], two leading high resolution next-generation sequencing-based technologies.

In Methyl-Seq [15], genomic DNAs from the same biological sample are digested by enzymes MspI and HpaII, respectively. MspI cleaves all 5′-CCGG-3′ sites; while HpaII cleaves only unmethylated 5′-CCGG-3′ sites. The digested fragments are then subject to size-selection, which acts to enrich the CpG-containing regions in the fragment library. Afterwards, the selected fragments are sequenced on the next-generation sequencing platform. Sequence tags in MspI digestions delineate “assayable” sites, while sequence tags in HpaII digestion identify unmethylated sites specifically. Thus the methylation level at each assayable site can be inferred by the presence or absence of HpaII tags. In RRBS, genomic DNAs are also first enriched for CpG contents by MspI digestion. However, the methylation status of sites is probed by bisulfite sequencing. Bisulfite treatment of DNA converts unmethylated cytosine nucleotides into uracils (and read out as ‘T’s), and the methylation status of a site can be inferred by comparing the sequence tag to the reference genomic sequence. Methyl-seq and RRBS technologies are different in the way methylation signals are measured. The Methyl-Seq performs methylation-specific digestion and thus only reads out signals at 5′-CCGG-3′ sites, while RRBS performs bisulfite sequencing which reads out signals at all cytosine nucleotide positions in the selected fragments.

Both Methyl-Seq and RRBS data involve methylation-sensitive tag counts and are likely to benefit from statistical methods for the quantification of methylation levels, rather than direct read counting. For Methyl-Seq, Brunner *et al.* [15] used the binary call of methylation level. However, since most experiments involve heterogeneous mixtures of tissues or cells with different methylation levels, ideally the methylation proportion
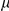
should be treated as a continuous variable between 0 and 1 that reflects the percentage of methylated molecules in the mixture of cells from which the DNA was sampled. Moreover, Brunner *et al.* [15] 's estimation is only based on HpaII tag counts whereas MspI tag counts are merely used to delineate “assayable” regions and HpaII tag counts are used to make a binary call. It would be reasonable that combining the tag count information of MspI and HpaII naturally contribute to proportion estimate in the methylation quantification. For RRBS, the natural quantification of methylation level at CpG dinucleotides would be the number of tags with C divided by the total number of tags. For both Methyl-Seq and RRBS, due to the random sampling nature of shotgun sequencing, the coverage at different sites varies and thus the variance of the estimates for the methylation level can be large and heterogeneous. It would be desirable in this sequence-based technology to estimate the variance of methylation level, which is potentially useful for further epigenetic association studies. Since the Methyl-Seq technology was developed very recently, there have been very few methods developed for statistical quantification for Methyl-Seq and RRBS data. Recently, the MetMap program developed by Singer *et al.* [17] infers site-specific methylation probabilities by a statistical graphic model. This program primarily focuses on the setting where paired-end HpaII fragment libraries without corresponding MspI libraries are sequenced, resembling the methylation sensitive cut counting approach [18]. In addition, the MetMap program infers strongly unmethylated islands with a hidden markov model like structure.

In this work, we study the statistical issues relating to the quantification of methylation levels by next-generation sequencing technologies: Methyl-Seq and RRBS. Since the quantification of RRBS is relatively straightforward, we mainly focus on Methyl-Seq. Unlike MetMap, we assume that both the MspI-digested and the HpaII-digested libraries are available, and we do not assume paired-end information. We present two new methods to quantify methylation levels for Methyl-Seq data: one maximum likelihood estimate and the other in a Bayesian hierarchical model framework. Our Bayesian method, based on a Poisson thinning process [19], can accommodate varying sequencing depth among different genomic regions. We compare the performances of our models with both simulated and real data.

In addition to algorithm development, we investigate a few experimental design questions regarding quantification of methylation levels in next-generation sequencing. We compare the site-level versus the region-level quantification. Moreover, we estimate the necessary sequencing depth, at which Methyl-Seq can offer a comparable quantification consistency as microarray. Finally, although the quantification for RRBS is more straightforward than Methyl-Seq, we reveal an important difference of the variances of these two technologies.

## Methods

### 2.1 Background on Methylation estimation in Methyl-Seq (Brunner *et al.*)

Using next-generation sequencing, Methyl-Seq assays over 250,000 methylation-sensitive restriction enzyme cleavage sites grouped into over 90,000 regions. In their original paper, Brunner *et al.* [15] demonstrated the Methyl-Seq technology by analyzing the methylation pattern for 13 human tissue types. In their experiments, one control sample of HCT116 tissue type was digested by MspI and 13 different tissue samples were digested by the methylation-sensitive enzyme HpaII. Because of some technical replicates, one MspI library and 15 HpaII libraries were generated (see Supplementary Table 2 of Brunner *et al.* [15] for details). These digested fragments undergo fragment size selection, and most fragments are of length 35–75 bps. Because the enzyme cleavage sites 5′-CCGG-3′ contain a CpG sites and CpG sites are known to be clustered, the size selection process will enrich the presence of CpG sites in the library. After size-selection, these libraries were subjected to next-generation sequencing, resulting approximately 3 million tags (sequencing reads) per HpaII library and 10 million tags for the MspI library.

The following bioinformatics processing was conducted to obtain tag counts at each digestion site. First, all reads were mapped to the reference human genome sequence. Not all CCGG sites in the human genome are covered by sequencing reads due to the fragment size selection and various sequencing biases. In practice, only those digestion sites that are covered by four or more MspI reads in either forward or backward direction were deemed as “assayable” sites. For assayable sites, the tag counts in both forward and backward directions for each library were recorded. We downloaded the tag count data from the Myers lab website at HudsonAlpha (http://myers.hudsonalpha.org/content/protocols.html). Since methylation levels at nearby sites are typically highly correlated, Brunner *et al.* [15] grouped digestion sites in neighboring 35–75 bps into a “region” and methylation levels were called at the region level. Brunner *et al.* [15] 's methylation estimate was binary: a region in a library is either methylated or unmethylated. Specifically, in a region containing *n* sites, they used the HpaII tag counts at the *i*-th site, which is defined as 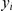. After grouping sites into previously determined assayable regions, each region's methylation level was called based on the average HpaII tag count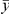. Regions with 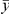 >1 were called unmethylated, the methylation level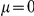; otherwise were called methylated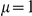.

To validate the Methyl-Seq technology, Brunner *et al.* [15] compared the Methyl-Seq tag counts with the results of the Infinium Human Methylation 27 BeadChips (Illumina), a standard technology for quantification of DNA methylation levels. For each of the CpG sites represented on this array, the beta value, calculated based on the intensities of the relevant probes, estimates the percent of DNA molecules being methylated. The comparison between Methyl-Seq and the microarray experiment was based on four tissue sample libraries: *HCT116, H9 hESC, H9 endoderm* and *adult liver* with overall 160 matching regions.

As a quantitative measure of the consistency between Methyl-seq and Infinium microarray, Brunner *et al*.[15] use the Receiver Operating Characteristic (ROC) curve. Basically, they dichotomize microarray beta values as the gold-standard (>0.6 as methylated and <0.6 as un-methylated), and consider average HpaII tag counts as the predictor. As a result, average HpaII tag counts in Methyl-Seq has an area under the ROC curve (AUC) 0.944 (Figure 1C in Brunner *et al*. [15]).

**Figure 1:**
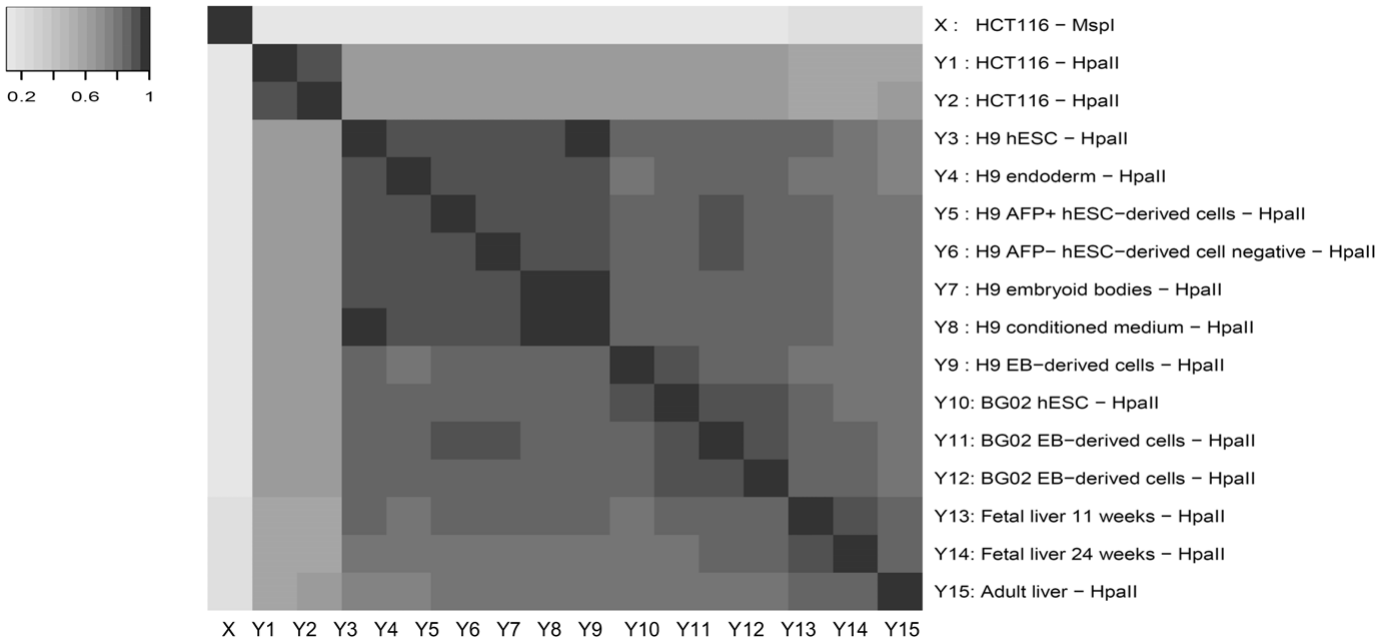
Correlation of 16 Tissue Sample Libraries. Spearman correlations among tag counts in MspI (X) library and 15 HpaII libraries (Y1–Y15).

### 2.2 Methyl-Seq Data Processing and Observations

In the present work, we use the Brunner *et al.* data set and discuss statistical issues relevant to Methyl-Seq. Due to this somewhat complicated experimental procedure, it may be beneficial to first review a few decisions made during the data processing, before presenting our observations on the issue of statistical quantification.

In a Methyl-Seq data set, there are two tag reads at each site, one on the forward strand and one on the reverse strand. While Brunner *et al.* used the larger of the forward and reverse tag counts, it can be tempting to use the tag counts for both forward and reverse reads. Ideally, If paired-end read libraries were used such as in [17], i.e., a pair of reads from both ends of a fragment, one on the forward strand and one on the reverse strand, are sequenced, it is possible to keep track of all fragments. However, when paired-end reads are not available it is not a simple problem to infer all fragment information. Moreover, under ideal conditions, the forward and the reverse tag counts at site *i* should be equal, as they both represent the digestion at that site. In reality, these forward and reverse counts may not be equal: it could simply be a reflection of the fact that site *i*-1 is further away from site *i* than site *i+1*, and it is sequenced less easily. In fact, when the next site is too far away, site *i* would only have reads from one strand. This gets even more complex with HpaII, as the distance to the next site is determined by the distance to the next unmethylated site. In the extreme, there will be no site for many kilobases, and the fragment will only be sequenced in one direction, so the forward reads will be present and the reverse reads absent (or nearly so). Therefore, for HpaII digestion, simply counting both forward and reverse reads will inflate the tag count at sites that are between two other nearby unmethylated sites. While a full treatment of the directionality of reads may be possible with a much more complicated model with explicit representation of fragments, we follow Brunner *et al.* [15] in this work and use the larger of the forward and reverse read counts.

Also, like Brunner *et al.* [15], we use the Infinium microarray experiment data as gold-standard reference. In addition to the microarray data used in Brunner *et al.* [15], we also use two new tissue sample libraries: *BG02 hESC* and *BG02 EB-derived cells*. We implemented a new background normalization procedure to the microarray data to improve the quantification. This involved subtracting the median of the negative control probes on each array from the red and green color channels, and recalculated methylation levels as b/(a+b), where b is the background-subtracted intensity from the methylated probe and a is the background-subtracted intensity from the unmethylated probe. We identified 151 regions in 6 tissue libraries matched between Methyl-Seq and microarray experiments. After eliminating 9 missing values within the newly generated microarray data, the comparison is based on 897 methylation beta values. The AUC of Methyl-Seq tag counts in our data set is 0.9556, slightly higher than that in the Brunner *et al,* [15] analysis.

To conduct a statistical analysis of the Methyl-Seq data, we define the following notations. For an assayable CCGG site *i*, we use *x_i_* denoting its MspI tag count and *y_i_* denoting its HpaII tag count. Following Brunner *et al*.[15], we use the larger of the forward and reverse tag counts at each site in a region. Also, we assume that all CCGG sites in a region have the same methylation level and we will quantify the methylation level for each region. In the present work we consider one HpaII library at a time, although there might be correlation of methylation levels among different libraries at the same site.

With this setup, we have the following observations on this data set. First, Brunner *et al*.[15] 's estimation only includes the HpaII tag counts information, whereas MspI tag counts are only used to delineate “assayable” regions. We understand that it is not the primary interest of Brunner *et al.* to give a continuous estimate of the methylation percent. However, with the MspI tag count information, it is possible to make proportional estimates between 0 and 1 for the Methyl-Seq data, which reflect the percentage of methylated molecules from which DNA was sampled. The naïve proportional estimate for the HapII library *j* would be 
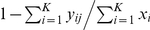
, where *K* is the total number of sites in a region.

Second, due to the random sampling nature of sequencing, for a given region in MspI and HpaII library sample *j*, the HpaII tag count 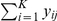 of all *K* sites is not always smaller than the MspI tag count 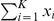. Therefore, the simple proportional estimate 
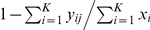
 may be negative and thus the estimate has to be revised.

Third, certain genome-wide correlation structures exist among the tag counts in these libraries (Figure 1). Primarily, the control Library (X: HCT 116 - MspI) digested with MspI has spearman correlation 0.11∼0.21 with libraries digested by HpaII 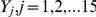. This correlation results from sequence-specific biases in the library construction process and the sequencing process, together with methylation effect. These effects are difficult to disentangle, but we can simply model them by introducing certain correlation between X and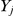. Besides, all pairs of libraries digested by HpaII show a high correlation (spearman correlation
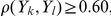
where *k, l* stands for any pair of HpaII libraries). This reflects the basal pattern of methylation that is unchanged among different cell types. Moreover, HpaII libraries from the same tissue samples (such as Y3–Y9 from H9) have generally an even higher correlation (spearman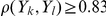), suggesting tissue-specific methylation profiles. Finally, technical replicates typically have the highest correlation. This is the case for Y1 and Y2 (spearman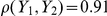), and for Y11 and Y12 (spearman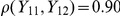).

To allow for a statistical analysis of the Methyl-Seq data, we explore some assumptions pertaining to the distribution of tag counts. Assuming genome-wide uniform sequencing depth, the MspI and HpaII tag counts along the genome can be approximated by Poisson distribution, where the tag counts' Poisson mean 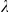 is the sequencing depth of MspI library [20]. However, since the sequencing depth is not constant throughout the whole genome, instead of using a constant depth parameter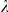 in Poisson distribution for the whole genome, our analysis uses dynamic sequencing depth parameter
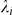
for each cleavage site *i*'*s* MspI library tag count: 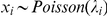
where 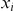 stands for the MspI library tag count for each cleavage site *i*. Moreover, Ji *et al.* [21] suggest that the ChIP-Seq tag counts can be better fitted with a negative-binomial distribution. A negative-binomial distribution can be modeled as a continuous Gamma-Poisson mixture structure [22], that is, we can fit 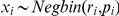 with the hierarchical model
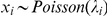
and the Poisson rate 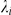 conditional on 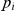 and 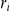 *:* 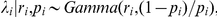 where 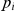 is a proportion parameter, and *r*_i_ is the over-dispersion parameter. In this way, Poisson assumption is a special case nested in the Negative-binomial assumption. Our analyses considered both assumptions and used the Gamma-Poisson mixture framework. We also define a constant Beta-value, the methylation level
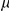
for each HpaII library in a specific region. Following Brunner *et al*.[15] 's analysis on restriction enzymes, each HpaII tag is an independent Bernoulli with parameter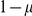. To estimate the methylation level 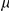, we propose two methods: Truncated Proportional Estimate (TPE), and Bayesian Hierarchical method. Both methods are detailed below.

### 2.3 Truncated Proportional Estimate

We assume the following model for generating the tag counts in Methyl-Seq experiment. For a region with K assayable CCGG sites, the HpaII tag count at the *i*-th site in *j*-th technical replicates, *y_ij_*, is generated by first generating *x_i_*' total sequencing tags, and then sub-sampled by a fraction (1-*μ*), where *μ* is the methylation level of the region. In another words, 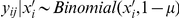, where 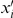
is the corresponding unobserved MspI tag count sample, generated from the same distribution as 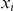: 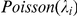 or 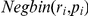. With either Poisson or more general negative-binomial assumption, we can derive
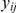
's marginal distribution respectively: 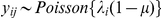 or 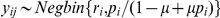. Regardless of the assumption of Poisson or negative binomial distribution of tag counts, we can use
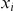
and
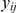
's log-likelihood to derive the same maximum likelihood estimate of *μ*: 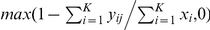. We called this estimate the Truncated Proportional Estimate (TPE): the term 
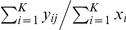
 estimate the proportion of unmethylated DNA and is consistent with the intuitive proportion between HpaII and MspI tag counts; when this term exceed one, methylation estimate is truncated to 0. TPE includes both MspI and HpaII sequence tag counts information as well as their random sampling nature into the estimation.

The TPE method is attractive since it is simple in calculation and does not depend on specific assumptions. However, this method cannot provide methylation levels' variance estimate. On the one hand, since sequencing coverage is not consistent among the whole genome, the variance of the methylation level can be large and heterogeneous. Therefore, estimation of the variance is often desired in association studies. On the other hand, based on Brunner *et al*.[15] 's reported sequencing data, 77% of all 90,612 regions in the whole genome are composed of only two digestion sites, and 95% of regions in the whole genome contain no more than 5 digestion sites. Therefore, the sample size for methylation estimate is small, and it may be not appropriate to use the observed information matrix [23] of Maximum Likelihood Estimation to approximate the estimates' variance. Moreover, because of the truncation toward 0 when 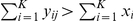, the proportional estimate is biased downward. To alleviate the lack of estimates' variance and the extreme bias at high HpaII count cases (low methylation), we consider a Bayesian Hierarchical model approach.

### 2.4 Bayesian Hierarchical Model

Bayesian hierarchical models have been successfully applied in modeling ChIP-Seq data [24] and RNA-Seq data [25], because they offer flexibility in modeling complex processes of generating sequencing tag counts. Moreover, Bayesian hierarchical models framework allow estimation of the posterior distribution of parameters, and therefore their variances.

With the Poisson assumption of tag counts, the MspI tag count 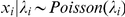, and each HpaII tag count is an independent Bernoulli with parameter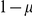, then the HpaII tag count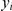 can be considered as the result of a Poisson thinning process [19], and is distributed with 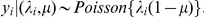 If we assume the negative-binomial model of tag counts and consider the Gamma-Poisson mixture, we can specify the distribution of the sequencing depth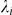 conditional on
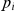
and 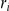: 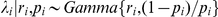 where
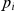
is a proportion with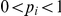, and 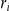 with 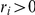 is over-dispersion parameter, which reflects the extra variance of 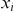 beyond the Poisson assumption. When 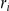 approaches infinity, the negative-binomial assumption is equal to the Poisson assumption. For the final level of the hierarchy, without any prior information of methylation level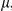 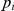
and 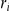, we use non-informative priors for these parameters. In summary, our hierarchical model is:



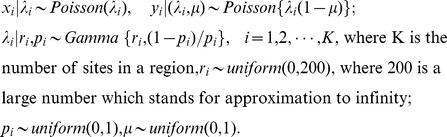



Then the joint posterior density of 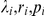
and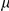 can be expressed as: 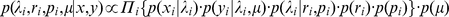



In order to compute the posterior estimates and variance of parameters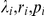 and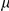, we use the Markov Chain Monte Carlo (MCMC) to generate posterior samples from the posterior distribution of the parameters. In the following analysis, we fit the Bayesian model using Winbugs software [26]. We ran three different chains from independent initial values, and specify 500 iterations as burn-in. After convergence, there are 500 iterations to generate posterior distribution for all parameters. Since there are overall 3*K+1* parameters to be estimated in the model and most of regions are composed of small number of cleavage sites *K*, the MCMC algorithm converges quickly. With the generated posterior samples, we can compute posterior mean as methylation estimate and its variance.

### 2.5 Flexible Structure to Adjust Sequencing Depth Bias

Within Brunner *et al*.[15] 's data, sequencing is performed generally deeper on MspI libraries than on HpaII libraries, and the bias is different between HpaII libraries and regions. In Brunner *et al*. 's original analysis, since methylation binary call only depends on the HpaII tag count, it is not a crucial problem. However, the sequencing depth bias affects MspI and HpaII libraries' tag counts differently, and it should be adjusted for the estimation of methylation level
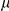
in our models. Lacking the bias information for each region, we use genome-wide CGG tags aligned to MspI sites (see Supplementary Table 2, last column, of Brunner *et al*.[15]) as the reference, and define the ratio of MspI library to each HpaII library to specify its sequencing depth bias 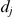. Recognizing that this ratio combines the methylation effect with sequencing depth bias, the adjustment is only approximate.

Both the Bayesian Hierarchical model and the TPE model provide flexible structures to adjust this known biases 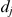. For instance, Bayesian method's hierarchical distributions change to: 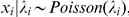 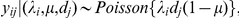 and other terms remain unchanged. Meanwhile, TPE of
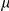
can also incorporate the sequencing depth bias, and changes to: 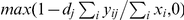.

## Results

### 3.1 Evaluation Quantification by Simulation Study

We use simulation studies to evaluate the proposed Bayesian estimate and the Truncated Proportional Estimate (TPE). We first generate a methylation level
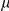
from the empirical density of Microarray beta value from Brunner *et al*.[15], and then generate cleavage site number *K* of each region based on Methyl-Seq real data's empirical distribution of sites. In this way, simulated data scenario is as similar to the real data example as possible. In the simulation, we assume that MspI tag counts are Poisson distributed, and design the sequencing depth to be a constant value, such as 50. Meanwhile, we consider the same sequencing depth of MspI and HpaII libraries, and thus there is no sequencing depth bias. We generate each site's MspI tag counts 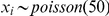, and HpaII tag counts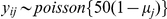, where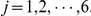 Overall, we simulated 6 tissue libraries' tag counts, across 155 regions, with total 930 methylation levels to be estimated. In addition, to compare the quantification by Methyl-Seq in different sequencing depths, we generated simulation data for 9 different sequencing depths increasing from 40 to 350.

**Figure 2:**
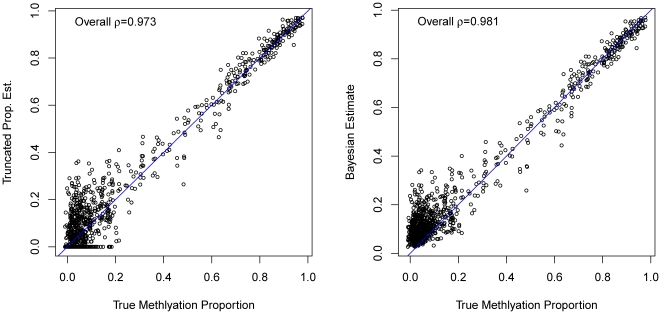
Performance of proposed estimates on simulation data at sequencing depth 50. Comparison of the proposed estimates (TPE and Bayesian Estimate) and the true methylation proportions in simulation. Bayesian estimate have significantly higher correlation (pearson 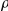 = 0.981) than TPE (pearson 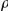 = 0.973) (p-value<0.001, t test).

We applied TPE and Bayesian estimation methods to simulation data. In Figure 2, we plot two different methods estimates with sequencing depth 50 vs. the true methylation levels. It is shown in the plots that TPE as well as Bayesian estimate has increasing variance as methylation level
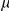
decreases. Moreover, TPE shows a prominent truncation at zero at low methylation level, which is not the case for Bayesian estimate. Overall, Bayesian Hierarchical estimate correlate with true methylation beta value better than TPE (t-test [27] for Pearson correlation differences 0.007, p-values <0.001).

As another way to compare different methods' estimates, we follow Brunner *et al.* [15] and create estimates' ROC curves. We compare the estimates of the methylation level by the Brunner *et al.* 's HpaII tag count, TPE, and Bayesian methods with dichotomized microarray beta values. Because of the dichotomization of microarray data, the ROC evaluation is not as sensitive as correlation analysis. Still, ROC serves as an alternative evaluation of quantification and a better estimation method should have a higher area under the ROC curve (AUC). What we found is that, consistent with the Pearson correlation result, Bayesian Hierarchical estimate slightly outperforms the truncated proportional estimate with a higher AUC (data not shown).

### 3.2 Evaluation of Quantification by Real Methyl-Seq Data

**Figure 3:**
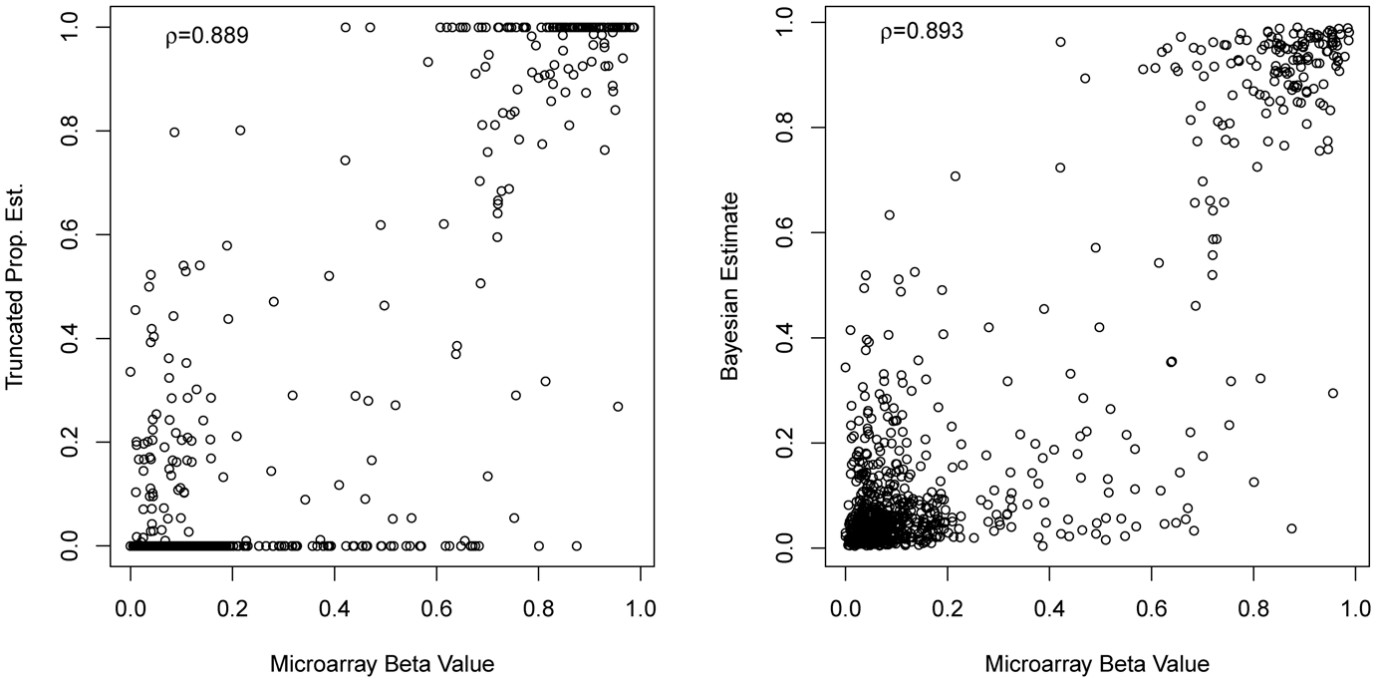
Performance of proposed estimates on Methyl-Seq real data. Comparison of the proposed estimates (TPE and Bayesian Estimate) in real Methyl-Seq experiment and microarray methylation beta values. Bayesian estimate has significantly higher correlations than TPE (p-value = 0.012, t test).

We next applied the Truncated Proportional Estimate (TPE) and Bayesian estimate to quantify the methylation levels in Brunner *et al.* [15] Methyl-Seq data set, which is introduced in Methods section 2.2. In addition, we use the adjustment in Methods section 2.5, with genome-wide CGG tags information to specify the overall library-wide sequencing depth as mentioned in Brunner *et al*.[15]. In Figure 3, we plot the TPE and the Bayesian estimates against the microarray beta value. For both estimation methods, most of data points cluster around lower-left (“low-low”) and upper-right (“high-high”) corners. This indicates that the Methyl-Seq estimates which coincide with microarray beta values usually occur in high methylation (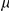
is close to 1) or low methylation levels (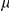
is close to 0). However, a notable fraction of Methyl-Seq estimates deviate from microarray beta values, visible on the plots of Figure 3 as off-diagonal points, reflecting that either of two estimates does not fit the microarray beta value as well as the simulation study. Overall, the Bayesian estimates achieve a correlation of 0.893 with the microarray beta values; while that correlation for the TPE method is 0.889. This difference is significant (t-test for correlation difference 4×10^−3^, p = 0.013).

While this overall high correlation levels reflect the fact that both TPE and Bayesian methods are capable of capturing the binary “high-low” classification of methylation levels, it is worthwhile to investigate the detailed quantification performance at “high-high” and “low-low” regions. In Figure 3, if we consider the “low-low” region with both the TPE and Bayesian estimates, as well as the microarray beta values all less than 0.5, the correlation of Bayesian estimate is 0.207, while the correlation of TPE is 0.158. On the other end, if we consider the “high-high” region with both the TPE and Bayesian estimates, as well as the microarray beta value greater than 0.5, the correlation of Bayesian estimate is 0.415, while the correlation of TPE is 0.416. Moreover, while TPE apparently truncated some points at zero, Bayesian method eliminated these truncated estimates.

In fact the difference of quantification of TPE and the Bayesian method is more pronounced than the overall correlation suggested as shown in Figure 3. TPE's estimates are sharply concentrated at extreme values: zero and one. This is because that the truncation acts in regions where HpaII tag counts exceed MspI tag counts and thus forces the methylation estimates to 0, and in regions devoid of HpaII tags and thus the methylation estimates are exactly at 1. The Bayesian estimates do not show such sharp truncations, and thus are more amicable for real-world applications.

**Figure 4:**
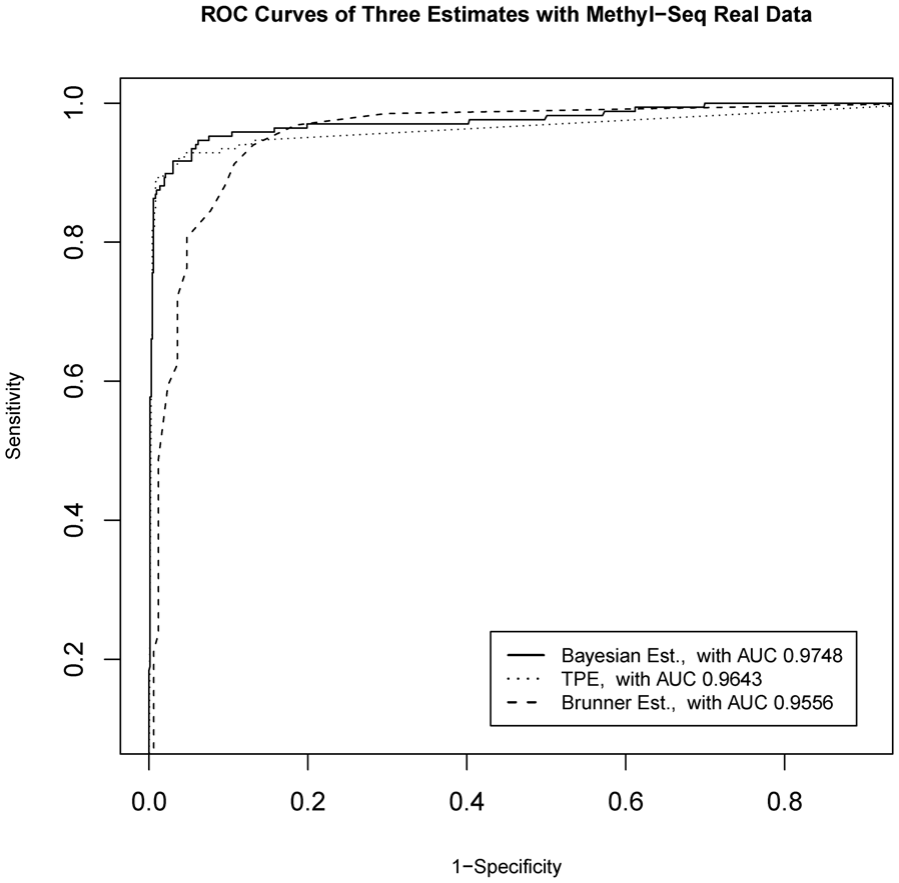
ROC curve Comparison of proposed estimates with Brunner *et al*. 's estimate. ROC curves for three quantification methods: Brunner Estimate (HpaII tag count), Truncated Proportional Estimate (TPE), and Bayesian Estimate. Following Brunner *et al.*, microarray beta values are treated as gold-standard and dichotomized with >0.6 for methylated and <0.6 for un-methylated. The Brunner, TPE, and Bayesian estimates are treated as predictors, and each point on the plot represents a cutoff values on the continuous-valued predictor.

It is also shown from the ROC curves comparison (Figure 4) that TPE and Bayesian estimate have generally overlapping ROC curves, and have a higher AUC than Brunner *et al*. 's HpaII tag count. Moreover, the Bayesian estimate has a slightly larger AUC than TPE.

### 3.3 Necessary Depth Required by Methyl-Seq to Offer a Comparable Quantification Accuracy as Microarrays

**Figure 5:**
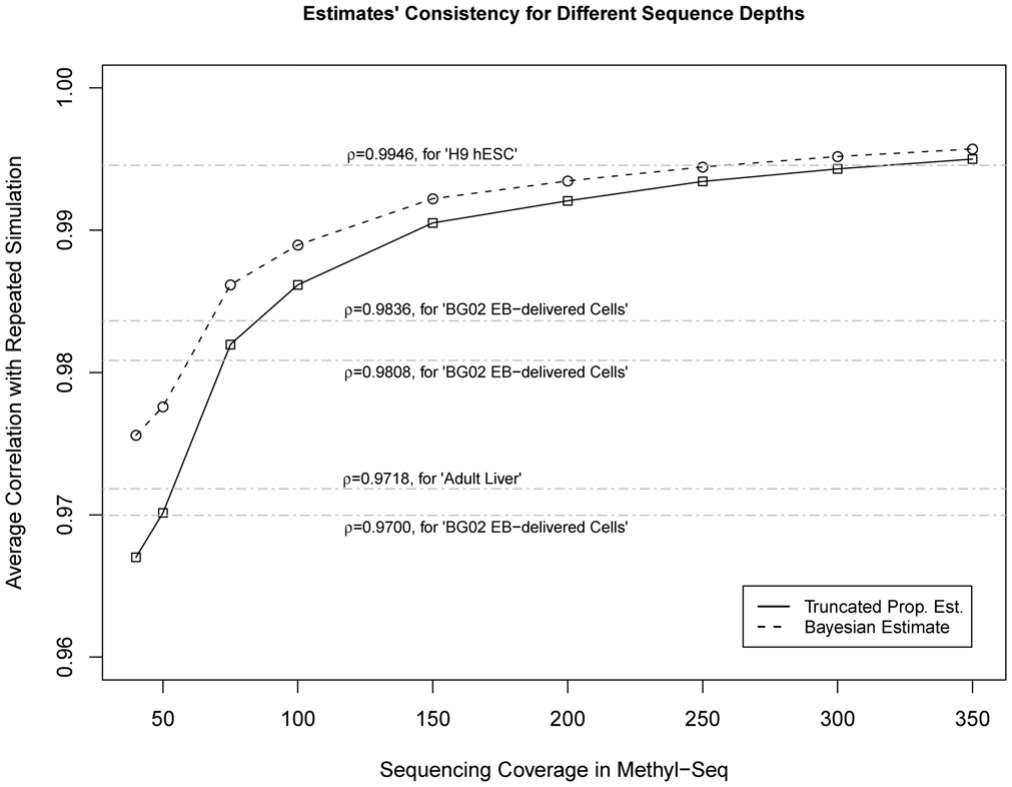
Quantification consistency of proposed estimates with increasing sequencing depth. Consistency of the proposed estimates for increasing sequencing depths in Methyl-Seq. Consistency is the average correlation between the measurements from technical replicates. Simulated technical replicates are generated from repeated sampling of the same underlying true methylation levels.

The estimate of methylation level in Methyl-Seq, as a count-based quantification, is more accurate with higher sequencing tag counts. A practical question is, at what sequencing depth can Methyl-Seq offer a better quantification than microarrays. We found 3 tissue sample libraries with technical replication data in microarray methylation experiments on the Infinium Methyl 27 platform: *H9 hESC* (2 replicates, with correlation 0.9946); *BG02 EB delivered cells* (3 replicates, with pairwise correlations 0.9836, 0.9808 and 0.9700); and *adult liver* (2 replicates with correlation 0.9718*)*. To achieve the same level of consistency of technical replicates with Methyl-Seq, one has to increase the sequencing depth. We simulate with different sequencing depths ranging from 40 to 350. It is clear from Figure 5 that the consistency (correlation) improves with increasing sequencing depth. It is also shown in Figure 5 that Bayesian method's correlation is always higher than TPE. To achieve a microarray's consistency, the Bayesian method needs sequencing depth about 40–250 per cleavage site while TPE would need 50–300. As a cautionary note, we remark that the correlation between repeated simulations' estimates is a measure of consistency, whereas the actual accuracy should be estimated by the correlation between the estimate and the true values, which is not yet available for our data set. Nonetheless the high accuracy shown in Figure 2 suggests that the consistency is a good estimate of the accuracy.

### 3.4 Site-level versus Region-level Quantification

A key advantage of Methyl-Seq/RRBS over ChIP-based methylation quantification technologies such as MeDIP [28] is that Methyl-Seq and RRBS can offer single base-pair resolution methylation status. We compare the site-level versus the region-level quantification using simulation. For simplicity, we only consider two sites in a region, and assume that MspI tag counts are fixed on sequencing depth, with a constant value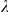. Meanwhile, we consider the same sequencing depth of MspI and HpaII libraries, and generate each site's MspI tag counts 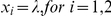, HpaII tag counts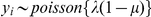. We first use the empirical density of microarray beta values [15] to fit the methylation level 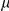 with a marginal beta distribution, resulting with 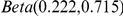, and then generate methylation level 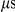 for two neighborhood sites with the same marginal distribution but different correlation [29], increasing from 0.92 to 1. Overall, we simulated 2000 sites for each correlation from 0.92 to 1. In addition, to compare the quantification in different sequencing depths, we generated simulation data with sequencing depths 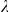 30 and 300.

**Figure 6:**
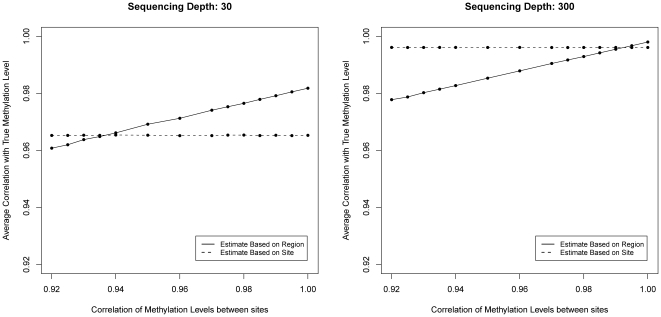
Comparison of performances of site level and region-level Bayesian estimations at high and low sequencing depths. The region-level estimation's accuracy increases rapidly with higher correlation, while the site-level estimation's accuracy remains stable. The region-level estimation has clear advantage for higher correlation region sites or lower sequencing depth, while the site-level estimation has better result for lower correlation region sites and higher sequencing depth.

As shown in Figure 6, we found that the site-level quantification is more accurate when the sequencing depth is high or the correlation of methylation levels between nearby sites is low. When sequencing depth is low and correlation of methylation levels between nearby sites is high, quantification by region will be more accurate than quantification by sites as it allows borrow information across all sites in a region. In many practical settings including the Brunner *et al.* [15] experiment, the sequencing depth is relatively low. Region-level quantification may be more appropriate in such cases.

### 3.5 Sensitivity of the estimators to different sequencing depths of MspI and HpaII libraries

A simplifying assumption in our analysis on Methyl-Seq data is that the sequencing depths in MspI library and HpaII library are equal, i.e., 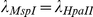. It would be helpful to know to what extent different depths are tolerated by the noise in the system. Here we investigate the effect of different sequencing depths in MspI and HpaII libraries on the estimate by simulation. We use the simulation procedure as described above in Results section 3.1 to simulate regions and sites, except that we generate 9 configurations of sequencing depths 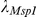 and 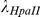. Specifically, we consider three different MspI sequencing depths: low (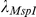 = 5), medium (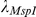 = 30), and high 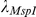 = 300). To control the level of sequencing depth discordance between the HpaII library and the MspI library, we generate the HpaII sequencing depth using log-normal distribution: 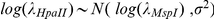. For each MspI setting we consider three levels of sequencing depth discordance with 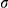 from 0, 0.5 to 1. For each combination of 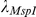 and 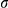 we generate 100 data sets, each with 6 tissue libraries' tag counts, across 155 regions, with total 930 methylation levels to be estimated. We run TPE and Bayesian model and compare the estimates to the true levels.

**Figure 7:**
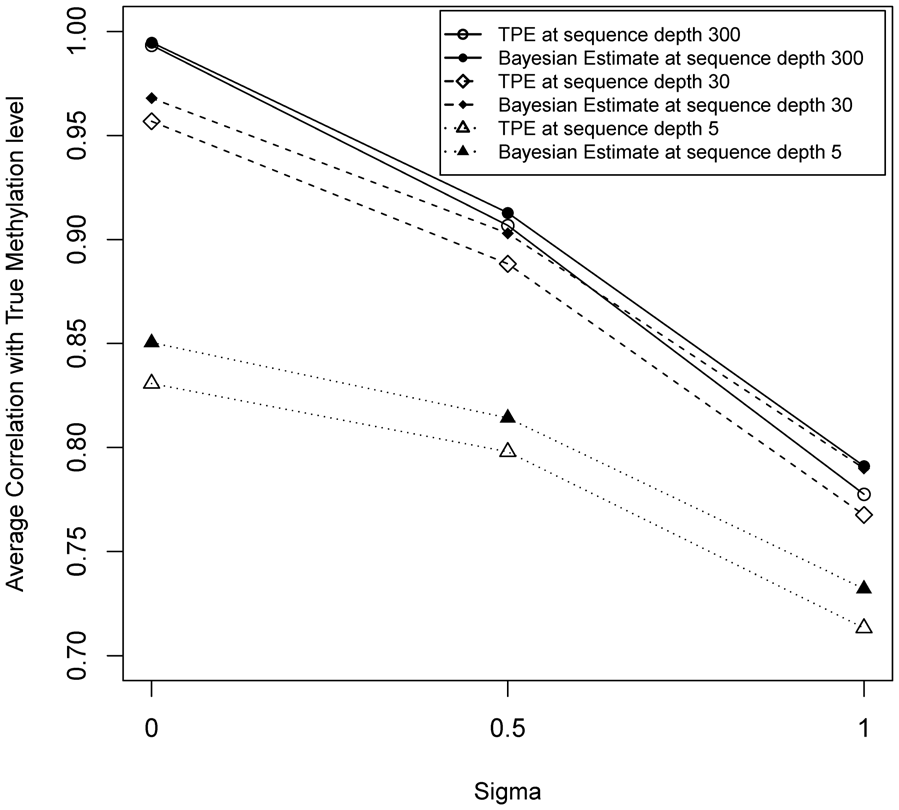
Effect of sequence depth difference between MspI and HpaII. Methyl-Seq assumes that MspI and HpaII sequencing tags share the same sequence depth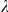. In many practical settings, the sequence depths are correlated but may not be the same. We consider the sequence depth difference with 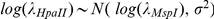, and compare the average correlation while the deviation 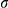 changes from 0, 0.5 to 1. Furthermore, we consider the sequence depths difference effect in three settings: low sequencing depth 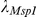 with 5, medium sequence depth 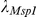 with 30, as well as extremely high sequencing depth 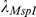 with 300. The sequence depth difference brings accuracy to drop rapidly. The effect of sequence depth difference is more heavy than the sequence depth.

As shown in Figure 7, we found that the quantification accuracy plummeted as the sequencing depth discordance increases: At sequencing depths of 30 or above, the correlation is above 95% when no discordance exist, while the correlation is about 90% with modest discordance (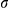 = 0.5), and the correlation is at 80% or lower when high discordance exists (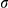 = 1). Meanwhile, the quantification with higher sequencing depth always helps. While there is a huge difference between low (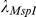 = 5) and medium (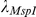 = 30) sequencing depths, additional sequencing depths above 30 seems only to increase correlation 2%–3%. Besides these patterns, it is also shown that the Bayesian estimates are consistently better than the Truncated Proportional Estimates.

In many practical settings, including Brunner *et al.* 's real data, the sequencing depths of the MspI and HpaII libraries are correlated but are not guaranteed to be the same, *ie*, there exists regional variations of sequencing depth in the experiment, even after the global library-wide sequencing depth is adjusted. While our model assumes no discordance between the sequence depths among libraries, our results suggest that the Bayesian model displays a higher level of robustness to this unknown noise than the naïve TPE model.

As a caveat, a scatter plot (Supplementary Figure S1) suggests that the heavy distribution of TPE estimates at extreme values (zero and one) in real data might be due to low sequencing depths in some regions.

### 3.6 Comparison of Methyl-Seq and RRBS in terms of the variance of their quantification

The quantification of RRBS data is relatively straightforward in the spirit of proportional estimate. For a site with a C nucleotide in the reference genome covered by MspI fragments, we denote the number of sequencing tags with ‘C’ at the site as *x*, and the number of sequencing tags with ‘T’ at the site as *y*, and the methylation level at the site would be simply *x*/(*x*+*y*). This site-level estimate can be generalized to region-level estimate as Σ*x_i_*/Σ(*x_i_*+*y_i_*), where the *x_i_* and *y_i_* are the sequencing tag counts of site *i* in the region.

**Figure 8:**
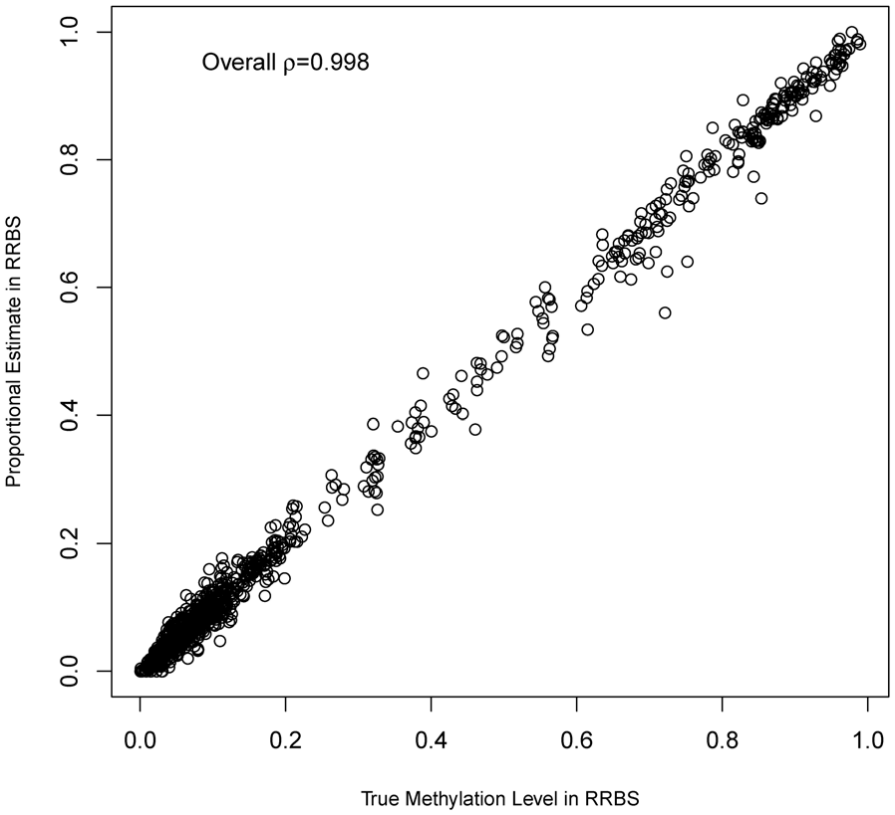
Performance of proposed estimates on RRBS simulation data. The RRBS data simulation is similar with Methyl-Seq data simulation. But we assume that 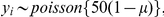
and 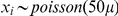. The Methylation level's variation keep consistent when μ = 0 and μ = 1, which is different from Methyl-Seq in Figure 2, where μ's variation inflates as μ decreases from 1 to 0.

Using simulation, we reveal, however, that Methyl-Seq's TPE and RRBS's proportional estimates have different behavior in terms of the variances of their estimates. For convenience of comparison, we apply the RRBS simulation procedure similar to Methyl-Seq in Results section 3.1, and simulate the regions, sites, and tag counts with the same sequencing depth 50 as in Methyl-Seq, except that we assume 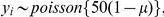 and 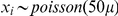. As shown in Figure 8, RRBS's estimates has a relatively equal level of variance near μ = 0 and μ = 1. This is in dramatic contrast to the Methyl-Seq's TPE estimate shown in Figure 2, where the variance is higher in the near μ = 0 range.

This reflects the nature of the data rather than the biases in these estimates. In Methyl-Seq, the MspI tag counts serve as a “control” of the HpaII tag counts. At sites where methylation level is low, the HpaII counts can be high but the MspI tag counts may be low and thus TPE truncation happens or the MspI tag counts may be very high and thus the proportional estimate can be quite different from the true methylation level. Noise of MspI tag counts in either direction can result in large deviation of TPE from the true value. At sites where methylatioin is high, the HpaII tag counts tend to be low, the variation of MspI tag counts would have a smaller effect on proportional estimates. In RRBS, the ‘C’ tag counts and the ‘T’ tag counts are symmetric and variances of the proportional estimate at either extremely high or extremely low methylation levels behave similarly. See Supplementary Part A in Materials S1 for a proof sketch for these arguments.

## Discussions

Methyl-Seq and other emerging sequencing-based technologies can measure DNA methylation levels in a sample efficiently. However, to the best of our knowledge, there is no investigation on the statistical issues related to the quantification of methylation levels in Methyl-Seq and other methylation sequencing data. In this study, we introduced two different methods for estimating the methylation levels for the Methyl-Seq technology: one intuitive Truncated Proportional Estimate (TPE) based on Maximum Likelihood estimation and the other in Bayesian hierarchical framework. Comparing these quantification methods through simulation and real Methyl-Seq data, we demonstrated that Bayesian hierarchical model outperforms the TPE, while both methods are significantly better than the binary quantification in the original Methyl-Seq paper [15]. This result indicates that the Bayesian hierarchical structure can effectively capture the statistical signals in the complex experimental design of Methyl-Seq. While TPE is conceptually simple and easy to implement, we recommend using Bayesian hierarchical structure as the statistical quantification method for Methyl-Seq.

In addition, we investigated several statistical issues relating to methylation quantification by sequencing. We found that, to achieve a quantification quality comparable to microarrays, Methyl-Seq should be conducted with at least sequencing depth 40–250 per cleavage site for both MspI and HpaII libraries. Also, using Bayesian method could save 15–20% in sequencing depth over using TPE to achieve the same level of quantification quality. Finally, we revealed an important difference between the variances of Methyl-Seq and RRBS: Methyl-Seq has an inflated variance for methylation level estimates at lowly methylated sites, while RRBS does not have such an artifact. All quantification methods for Methyl-Seq and RRBS have been implemented in an R-package, msBayes, freely available at http://www.ssg.uab.edu/wiki/display/SQML/Home.

There are additional biases in the Methyl-Seq data that have not yet been adjusted in our models. First, since methylation status and sequencing depth are coupled in the HpaII library, the overall library-wide sequencing depth might be underestimated by Brunner *et al.* by adjusting it with genome-wide CGG tags aligned to MspI sites [15]. While we followed Brunner *et al.* 's procedure, one possible future improvement is to iteratively re-adjust the overall library-wide sequencing depth after the quantification of methylation levels. Second, a major confounding factor for methyl-sequencing data is that the read frequency for a specific restriction site depends not only on the DNA methylation status at this particular site, but also on the DNA methylation status of neighboring sites. This is because, in order to obtain an HpaII read at site *i*, there has to be another HpaII cleavage site not too far from site *i* to present a fragment for sequencing. Third, the regional sequencing coverage λ_i_ is associated with many factors such as GC content, a common issue faced by many other sequencing-based technologies. It would be interesting to borrow ideas from other sequencing-based technologies such as RNA-Seq. For example, we can apply the Poisson log linear regression in our Bayesian hierarchical structure to model the sequencing preference by predicting λ_i_ from local sequences [30]. Fourth, in the context of Methyl-Seq and RRBS, an additional complicating factor is the selection bias of enzyme-cleaved fragments with different lengths (Supplemental Figure 4 of Brunner *et al.* [15]). The lengths of these fragments are associated with the regional density of 5′-CCGG-3′ sites. Our Bayesian model might be improved by incorporating components adjusting these biases and addressing these biases will be topics for future research. Fifth and finally, fragment size selection is an important source of sequencing depth bias, as shown in Supplemental Figure 4 in Brunner *et al.* Also, the variance/range of fragment sizes could influence the definition of regions in our quantification. As a background model, a restriction enzyme which is not only non-methylation dependent but also non-GC rich might be interesting to study as it teases out many sequence-dependent fragment selection biases.

In the present work, we follow the definition of regions by Brunner *et al.* [15]. Region definition is important as our models assume that methylation levels within a region remain a constant. We recognize that this region definition is rather simplistic. It is known that methylation levels can fluctuate even between nearby sites. More flexible constraints on the auto-correlation of methylation levels among neighboring sites may be explored as additional hierarchies in the Bayesian framework in the future. For example, one may incorporate a correlation matrix among neighboring sites.

The Reduced Representation Bisulfite Sequencing (RRBS) is an alternative sequencing-based technology for methylation quantification [16]. Similar to Methyl-Seq, RRBS is also a count-based sequencing technology, using restriction enzymes to recognize 5′-CCGG-3′ sites for enrichment of CpG sites. Unlike Methyl-Seq, RRBS uses bisulfite conversion technology and obtains both the tag counts for methylated and unmethylated DNAs from one tissue sample. However, RRBS may have distinct sources of biases such as the noisy alignment due to a reduced genome alphabet and noisy base calling at the first position of the fragment. Current quantification frameworks of both the TPE and the Bayesian Hierarchical model can be extended to quantify the RRBS data, with the Bayesian model is more promising in terms of handling the biases from diverse sources.

## Supporting Information

Figure S1
**Performance of proposed estimates on simulation data at low sequencing depth.** TPE and Bayesian estimates of methylation levels in simulation data generated using low sequencing depth (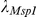 = 5). Please see Results section 3.5 in the main text for detailed simulation procedure. By visual comparison with Figure 3 in the main text, this result suggests that the extreme TPE estimates (zeros and ones) in the real data might be due to the setting of low sequencing depth.(TIFF)

Materials S1 (DOC)
