# Supplementary figures and images for: Statistical Quantification of Methylation Levels by Next-Generation Sequencing
Source: PLoS One. 2011 Jun 15;6(6):e21034. doi: 10.1371/journal.pone.0021034 (PMC3115964; doi:10.1371/journal.pone.0021034)

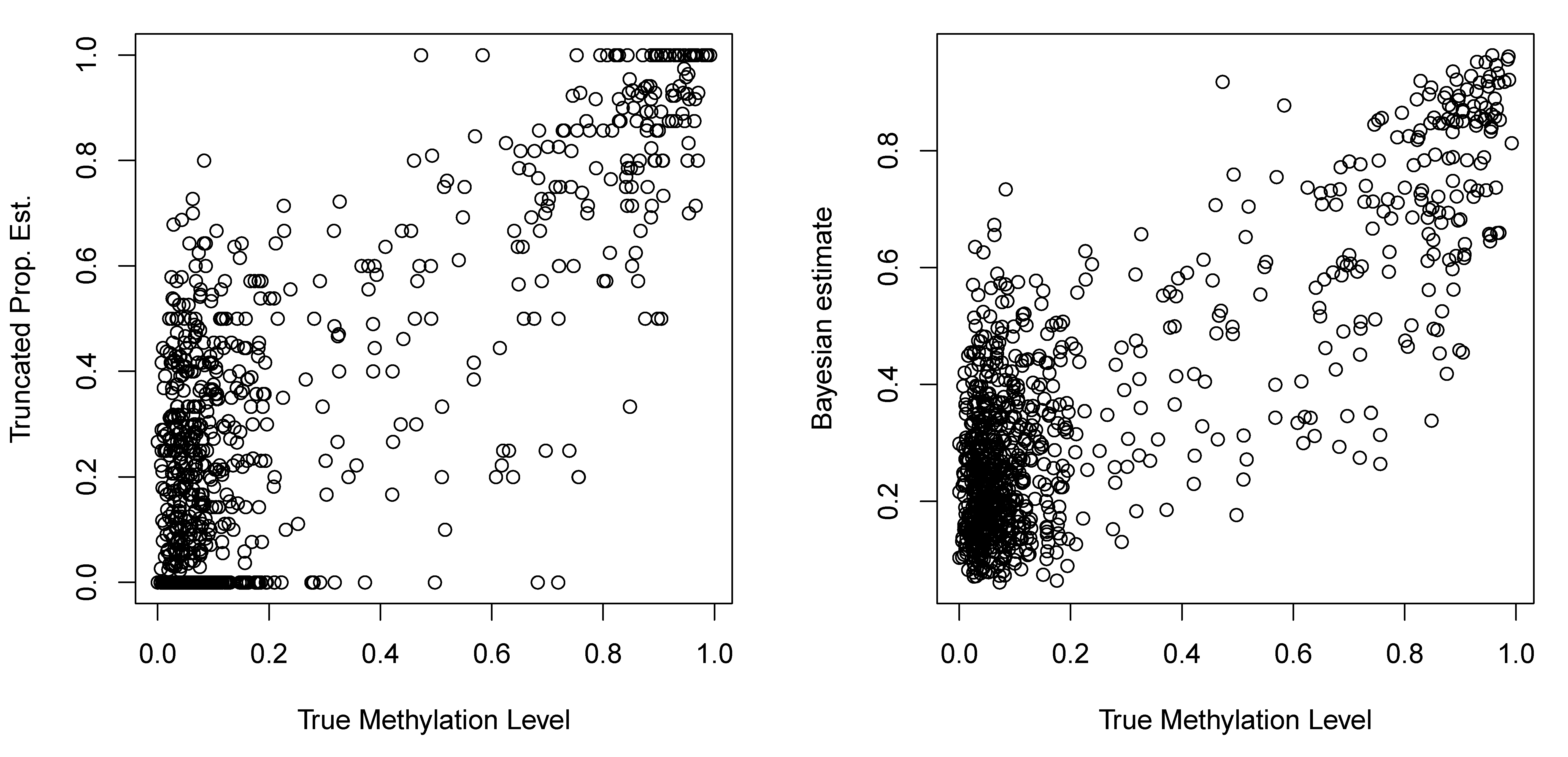

Supplement: Figure S1 — Performance of proposed estimates on simulation data at low sequencing depth. TPE and Bayesian estimates of methylation levels in simulation data generated using low sequencing depth ( = 5). Please see Results section 3.5 in the main text for detailed simulation procedure. By visual comparison with Figure 3 in the main text, this result suggests that the extreme TPE estimates (zeros and ones) in the real data might be due to the setting of low sequencing depth. (TIFF) [file pone.0021034.s001.tif]
