# Supplementary material for: Statistical Quantification of Methylation Levels by Next-Generation Sequencing
Source: PLoS One. 2011 Jun 15;6(6):e21034. doi: 10.1371/journal.pone.0021034 (PMC3115964; doi:10.1371/journal.pone.0021034)
Supplement: Materials S1 — (DOC) [file pone.0021034.s002.doc]

**Supplementary materials for**

**Statistical Quantification of Methylation Levels by Next-generation Sequencing**

Guodong Wu^1^, Nengjun Yi^1^, Devin Absher^2^, Degui Zhi^1^

^1^ Department of Biostatistics, University of Alabama at Birmingham, Birmingham, AL, USA

^2^ HudsonAlpha Institute for Biotechnology, Huntsville, AL, USA

**Part A**

***Claim:* In Methyl-Seq, when methylation level estimation is not truncated, *ie*** and, **methylation level estimation’s variance is increasing as methylation level $\mu$ decreases from 1 to 0.**

Given$x_{i}\sim Poisson(\lambda_{i})$and$y_{i}\sim Poisson\{\lambda_{i}(1-\mu)\}$, then variance of :is increasing as$\mu$decreases.

Proof: For random variable$A$and, with Tylor series approximation for quotient variance[1],

$$Var\left(\frac{A}{B}\right)=\frac{\left[E(A)\right]^{2}}{\left[E(B)\right]^{2}}\left\{\frac{Var\left(A\right)}{\left[E(A)\right]^{2}}+\frac{Var\left(B\right)}{\left[E(B)\right]^{2}}-\frac{2Cov\left(A,B\right)}{E(A)E(B)}\right\}$$

Thus if$\hat{\mu}\neq0$,$Var(\hat{\mu}_{j})=\mathrm{var}\left(1-\frac{\sum_{i} y_{i}}{\sum_{i} x_{i}}\right)$, since $x_{i}$and$y_{i}$ are independent, so $Cov\left(\sum_{i}y_{i},\sum_{i}x_{i}\right)=0$

$$\approx\left(1-\mu\right)^{2}\left\{\frac{1}{\sum_{i}\lambda_{i}}+\frac{1}{\sum_{i}\lambda_{i}(1-\mu)}\right\}=\frac{1}{\sum_{i}\lambda_{i}}(1-\mu)(2-\mu)$$

When$\mu$decreases, variance of $\hat{\mu}$ keeps increasing. The property is also true for Negative-binomial assumption, with similar proof.

***Claim:* In RRBS, methylation level estimation’s variance reach its maximum at methylation level $\mu$=0.5.**

Given$x_{i}\sim Poisson(\lambda_{i}\mu)$and$y_{i}\sim Poisson\{\lambda_{i}(1-\mu)\}$, then variance of :$\sum_{i}x_{i}/(\sum_{i}x_{i}+\sum_{i}y_{i})$ reach its maximum at methylation level$\mu$=0.5.

Proof: Similar to proof for Methyl-Seq,

$Var(\hat{\mu}_{j})=\mathrm{var}\left(\frac{\sum_{i}x_{i}}{\sum_{i}x_{i}+\sum_{i}y_{i}}\right)$, since $Cov\left(\sum_{i}x_{i},(\sum_{i}x_{i}+\sum_{i}y_{i})\right)=var(\sum_{i}x_{i})=\sum_{i}\lambda_{i}\mu$

$$\approx\left(1-\mu\right)^{2}\left\{\frac{1}{\sum_{i}\lambda_{i}}+\frac{1}{\sum_{i}\lambda_{i}\mu}-\frac{2\sum_{i}\lambda_{i}\mu}{\sum_{i}\lambda_{i}\mu\times\sum_{i}\lambda_{i}}\right\}=\frac{1}{\sum_{i}\lambda_{i}}\mu(1-\mu)$$

then variance of reach its maximum at methylation level$\mu$=0.5. The property is also true for Negative-binomial assumption, with similar proof.

Based on above approximate inference, proportional estimate’s approximate variance in RRBS: $\frac{1}{\sum_{i}\lambda_{i}}\mu(1-\mu)$ is always smaller than the un-truncated Methyl-Seq proportional estimate’s variance: $\frac{1}{\sum_{i}\lambda_{i}}(1-\mu)(2-\mu)$.

**References:**

1 Mood A.M. GFA, Boes D.C. : Introduction to the theory of statistics, ed 3rd McGraw-Hill Companies, 1974.

**Part B:**

**Figure S1: Performance of proposed estimates on simulation data at low sequencing depth**

**TPE and Bayesian estimates of methylation levels in simulation data generated using low sequencing depth ($\lambda_{MspI}$=5). Please see Results section 3.5 in the main text for detailed simulation procedure. By visual comparison with Figure 4 in the main text, this result suggests that the extreme TPE estimates (zeros and ones) in the real data might be due to the setting of low sequencing depth.**
